# Triclinic modification of diaqua­bis­(5-carb­oxy-1*H*-imidazole-4-carboxyl­ato-κ^2^ *N* ^3^,*O* ^4^)iron(II)

**DOI:** 10.1107/S1600536812031753

**Published:** 2012-07-21

**Authors:** Eriko Ohshima, Kazuki Yoshida, Kazumasa Sugiyama, Hidehiro Uekusa

**Affiliations:** a Department of Chemical Engineering, Ichinoseki National College of Technology, Takanashi, Hagisyo, Ichinoseki 021-8511, Japan; b Institute for Materials Research, Tohoku University, 2-1-1 Katahira, Aoba-ku, Sendai 980-8577, Japan; c Department of Chemistry and Materials Science, Tokyo Institute of Technology, O-okayama, Meguro-ku, Tokyo 152-8551, Japan

## Abstract

The title compound, [Fe(C_5_H_3_N_2_O_4_)_2_(H_2_O)_2_], is a triclinic modification of a monoclinic form recently reported by Du *et al.* [*Acta Cryst.* (2011) ▶, E**67**, m997]. The Fe^II^ ion lies at an inversion center and is coordinated by two N and two O atoms from two 5-carb­oxy-1*H*-imidazole-4-carboxyl­ate ligands in *trans* positions, together with two water mol­ecules, completing a slightly distorted octahedral coordination. Inter­molecular N—H⋯O hydrogen bonding between the N—H group of the imidazole ring and the deprotonated carboxyl­ate group builds a chain of 5-carb­oxy-1*H*-imidazole-4-carboxyl­ate anions along the [101] direction. The water molecules form intermolecular hydrogen bonds to O—C and O=C sites of the carboxylate group in adjacent layers.

## Related literature

For the structural diversity of the coordination architecture of the metal complexes of 4,5-imidazole­dicarb­oxy­lic acid, see Shimizu *et al.* (2004 ▶); Fang & Zhang (2006 ▶). For the isotypic Co analog, see: Li *et al.* (2011 ▶). For the coexisting phase, see Yakubovich *et al.* (1995 ▶). For the monoclinic form, see: Du *et al.* (2011 ▶).
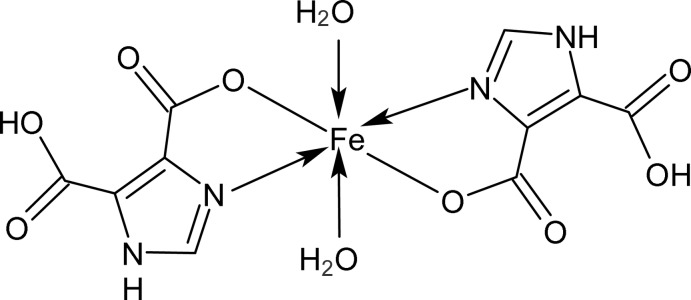


## Experimental

### 

#### Crystal data

[Fe(C_5_H_3_N_2_O_4_)_2_(H_2_O)_2_]
*M* *_r_* = 402.08
Triclinic, 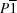

*a* = 4.9290 (5) Å
*b* = 6.4258 (6) Å
*c* = 12.2812 (10) Å
α = 78.161 (3)°
β = 85.175 (3)°
γ = 72.776 (4)°
*V* = 363.52 (6) Å^3^
*Z* = 1
Mo *K*α radiation
μ = 1.10 mm^−1^
*T* = 298 K
0.15 × 0.13 × 0.10 mm

#### Data collection

Rigaku R-AXIS RAPID diffractometer
Absorption correction: empirical (using intensity measurements) (*ABSCOR*; Higashi, 1995 ▶) *T*_min_ = 0.852, *T*_max_ = 0.898
3655 measured reflections
1668 independent reflections
1066 reflections with *I* > 2σ(*I*)
*R*_int_ = 0.050

#### Refinement

*R*[*F* ^2^ > 2σ(*F* ^2^)] = 0.041
*wR*(*F* ^2^) = 0.075
*S* = 0.92
1668 reflections
122 parameters
H atoms treated by a mixture of independent and constrained refinement
Δρ_max_ = 0.30 e Å^−3^
Δρ_min_ = −0.34 e Å^−3^

### 

Data collection: *RAPID-AUTO* (Rigaku, 1998 ▶); cell refinement: *RAPID-AUTO*; data reduction: *RAPID-AUTO*; program(s) used to solve structure: *SIR97* (Altomare *et al.*, 1999 ▶); program(s) used to refine structure: *SHELXL97* (Sheldrick, 2008 ▶); molecular graphics: *ORTEP-3 for Windows* (Farrugia, 1997 ▶); software used to prepare material for publication: *SHELXL97*.

## Supplementary Material

Crystal structure: contains datablock(s) I, global. DOI: 10.1107/S1600536812031753/fj2571sup1.cif

Structure factors: contains datablock(s) I. DOI: 10.1107/S1600536812031753/fj2571Isup2.hkl

Supplementary material file. DOI: 10.1107/S1600536812031753/fj2571Isup6.mol

Additional supplementary materials:  crystallographic information; 3D view; checkCIF report

## Figures and Tables

**Table 1:** Hydrogen-bond geometry (Å, °)

*D*—H⋯*A*	*D*—H	H⋯*A*	*D*⋯*A*	*D*—H⋯*A*
O5—H1*W*⋯O3^i^	0.86 (3)	1.86 (3)	2.705 (3)	165 (3)
O5—H2*W*⋯O4^ii^	0.79 (3)	1.95 (3)	2.702 (3)	158 (3)
N2—H2*A*⋯O1^iii^	0.86	1.95	2.767 (3)	157
O2—H2⋯O3	0.82	1.85	2.665 (3)	174

Symmetry codes: (i) 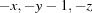; (ii) 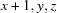; (iii) 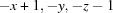.

**Fig. 1.**
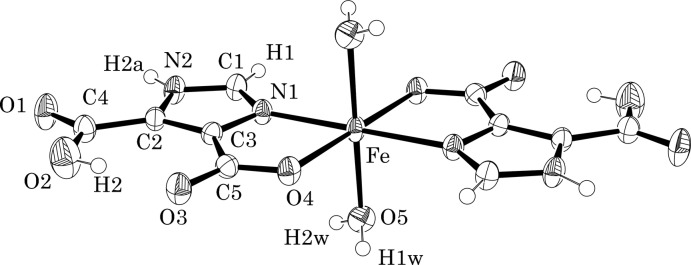
The molecular structure with displacement ellipsoids drawn at the 50% probability level.

## supplementary crystallographic information

### Comment

A number of studies on transition metal complexes with carboxylate ligands are reported in the literature. Recently, imidazole dicarboxylate has been recognized as an efficient building block, since it shows two different coordination modes to bridge or chelate metals through the carboxyl oxygen atoms and heterocyclic nitrogen donor. The crystal of diaquabis(5-carboxy-1*H*-imidazole-4-carboxylato-κ^2^*N*^3^,*O*)iron(II) is isostructural with Co analog 4,5-dicarboxyimidazole complexes (Li *et al.*, 2011). Iron(II) ion lies at the inversion center that is coordinated by two 1*H*-imidazole-4,5-dicarboxylate monoanionic ligands at the *trans* positions and two water molecules in a distorted octahedral geometry. A previous report described a monoclinic metal complex with a similar chemical composition (Du *et al.*, 2011). This structural diversity is attributed to the different types of coordination architectures of hydrogen bonding between molecules. The intermolecular hydrogen bonding between the N—H site of an imidazole ring and C═O site of a deprotonated carboxylate in the title compound builds a unique chain of 5-carboxy-1*H*- imidazole-4-carboxylate anion. The chain structures are further linked into a three-dimensional supermolecular framework through O—H···O hydrogen bonding interactions. The average distance of Fe—O agrees well with that of the monoclinic phase. Nevertheless, the average distance of Fe—N (2.165 Å) is longer than that in the monoclinic analog (2.147 Å).

### Experimental

A mixture of FeSO_4_. 7H_2_O (13.33 mmol), 4,5-imidazoledicarboxamide (22.21 mmol), 85.0% H_3_PO_4_ (8.89 mmol), and H_2_O (8 ml) was placed in a 30 ml Teflon beaker. It was sealed in a stainless-steel reactor, heated to 453 K for 96 h under autogenous pressure, and then, slowly cooled to room temperature. Pale-yellow block crystals of the title complex were isolated, washed with distilled water, and dried in air. It may be added that NH_4_FePO_4_.H_2_O: see Yakubovich *et al.* (1995), Pale-green plate crystals were also crystallized in the present synthetic condition. This supports the hydrolysis of the 4,5-imidazoledicarboxamide during the synthesis.

### Refinement

H atoms attached to C and N atoms were placed at calculated positions (C—H = 0.93 Å, N—H = 0.86 Å) and refined as riding atoms with *U*_iso_(H) = 1.2*U*_eq_(C, N). The carboxy H was located at the idealized position (O—H = 0.82 Å) and refined as a riding atom with *U*_iso_(H) = 1.5 *U*_eq_(O). On the other hand, H atoms of water molecules were located in a difference map, and their positions were subsequently refined with *U*_iso_(H) = 1.5*U*_eq_(O).

### Crystal data

**Table:**

[Fe(C_5_H_3_N_2_O_4_)_2_(H_2_O)_2_]	*Z* = 1
*M**_r_* = 402.08	*F*(000) = 204
Triclinic, *P*1	*D*_x_ = 1.837 Mg m^−^^3^
Hall symbol: -P 1	Mo *K*α radiation, λ = 0.71069 Å
*a* = 4.9290 (5) Å	Cell parameters from 3918 reflections
*b* = 6.4258 (6) Å	θ = 3.4–30.5°
*c* = 12.2812 (10) Å	µ = 1.10 mm^−^^1^
α = 78.161 (3)°	*T* = 298 K
β = 85.175 (3)°	Block, yellow
γ = 72.776 (4)°	0.15 × 0.13 × 0.10 mm
*V* = 363.52 (6) Å^3^	

### Data collection

**Table:**

Rigaku R-AXIS RAPID diffractometer	1668 independent reflections
Radiation source: fine-focus sealed tube	1066 reflections with *I* > 2σ(*I*)
Graphite monochromator	*R*_int_ = 0.050
Detector resolution: 100 pixels mm^-1^	θ_max_ = 27.5°, θ_min_ = 3.4°
ω scans	*h* = −6→6
Absorption correction: empirical (using intensity measurements) (*ABSCOR*; Higashi, 1995)	*k* = −8→8
*T*_min_ = 0.852, *T*_max_ = 0.898	*l* = −15→15
3655 measured reflections	

### Refinement

**Table:**

Refinement on *F*^2^	Primary atom site location: structure-invariant direct methods
Least-squares matrix: full	Secondary atom site location: difference Fourier map
*R*[*F*^2^ > 2σ(*F*^2^)] = 0.041	Hydrogen site location: inferred from neighbouring sites
*wR*(*F*^2^) = 0.075	H atoms treated by a mixture of independent and constrained refinement
*S* = 0.92	*w* = 1/[σ^2^(*F*_o_^2^) + (0.0339*P*)^2^] where *P* = (*F*_o_^2^ + 2*F*_c_^2^)/3
1668 reflections	(Δ/σ)_max_ < 0.001
122 parameters	Δρ_max_ = 0.30 e Å^−^^3^
0 restraints	Δρ_min_ = −0.34 e Å^−^^3^

### Special details

**Table:**

Geometry. All e.s.d.'s (except the e.s.d. in the dihedral angle between two l.s. planes) are estimated using the full covariance matrix. The cell e.s.d.'s are taken into account individually in the estimation of e.s.d.'s in distances, angles and torsion angles; correlations between e.s.d.'s in cell parameters are only used when they are defined by crystal symmetry. An approximate (isotropic) treatment of cell e.s.d.'s is used for estimating e.s.d.'s involving l.s. planes.
Refinement. Refinement of *F*^2^ against ALL reflections. The weighted *R*-factor *wR* and goodness of fit *S* are based on *F*^2^, conventional *R*-factors *R* are based on *F*, with *F* set to zero for negative *F*^2^. The threshold expression of *F*^2^ > σ(*F*^2^) is used only for calculating *R*-factors (gt) *etc*. and is not relevant to the choice of reflections for refinement. *R*-factors based on *F*^2^ are statistically about twice as large as those based on *F*, and *R*-factors based on ALL data will be even larger.

### Fractional atomic coordinates and isotropic or equivalent isotropic displacement parameters (Å^2^)

**Table:**

	*x*	*y*	*z*	*U*_iso_*/*U*_eq_	
Fe1	0.0000	0.0000	0.0000	0.0224 (2)	
O5	0.3576 (5)	−0.2580 (4)	0.06771 (17)	0.0314 (5)	
H1W	0.297 (7)	−0.370 (6)	0.097 (2)	0.047*	
H2W	0.489 (7)	−0.278 (6)	0.026 (3)	0.047*	
N1	0.1698 (5)	−0.0023 (4)	−0.16828 (18)	0.0237 (5)	
C1	0.3247 (6)	0.0920 (5)	−0.2439 (2)	0.0262 (7)	
H1	0.4099	0.1967	−0.2323	0.031*	
N2	0.3439 (5)	0.0184 (4)	−0.33916 (18)	0.0277 (6)	
H2A	0.4346	0.0598	−0.3986	0.033*	
C2	0.1936 (6)	−0.1351 (5)	−0.3258 (2)	0.0233 (7)	
C3	0.0869 (6)	−0.1468 (4)	−0.2178 (2)	0.0216 (6)	
C4	0.1805 (6)	−0.2443 (5)	−0.4200 (2)	0.0279 (7)	
O1	0.3050 (5)	−0.2040 (4)	−0.50906 (15)	0.0409 (6)	
O2	0.0250 (6)	−0.3870 (4)	−0.40319 (18)	0.0551 (7)	
H2	−0.0479	−0.3917	−0.3405	0.083*	
C5	−0.0966 (6)	−0.2761 (5)	−0.1514 (2)	0.0236 (6)	
O3	−0.1770 (4)	−0.4089 (3)	−0.19419 (15)	0.0328 (5)	
O4	−0.1626 (4)	−0.2417 (3)	−0.05321 (14)	0.0262 (5)	

### Atomic displacement parameters (Å^2^)

**Table:**

	*U*^11^	*U*^22^	*U*^33^	*U*^12^	*U*^13^	*U*^23^
Fe1	0.0257 (4)	0.0281 (4)	0.0170 (3)	−0.0135 (3)	0.0060 (3)	−0.0062 (3)
O5	0.0304 (14)	0.0328 (14)	0.0320 (12)	−0.0157 (12)	0.0077 (9)	−0.0019 (10)
N1	0.0283 (14)	0.0267 (14)	0.0211 (11)	−0.0140 (12)	0.0014 (10)	−0.0070 (10)
C1	0.0309 (18)	0.0317 (18)	0.0225 (14)	−0.0184 (15)	0.0019 (13)	−0.0067 (13)
N2	0.0314 (15)	0.0371 (16)	0.0195 (11)	−0.0195 (13)	0.0077 (10)	−0.0058 (11)
C2	0.0284 (17)	0.0243 (17)	0.0194 (14)	−0.0103 (14)	0.0006 (12)	−0.0056 (12)
C3	0.0225 (16)	0.0235 (16)	0.0191 (13)	−0.0078 (14)	0.0012 (11)	−0.0034 (12)
C4	0.0343 (19)	0.0304 (18)	0.0244 (16)	−0.0168 (16)	0.0028 (13)	−0.0077 (13)
O1	0.0533 (16)	0.0563 (16)	0.0234 (11)	−0.0307 (14)	0.0124 (11)	−0.0134 (11)
O2	0.073 (2)	0.0626 (18)	0.0436 (14)	−0.0405 (16)	0.0158 (13)	−0.0184 (14)
C5	0.0250 (17)	0.0234 (17)	0.0239 (15)	−0.0083 (14)	−0.0014 (12)	−0.0052 (13)
O3	0.0458 (14)	0.0345 (13)	0.0281 (10)	−0.0261 (12)	0.0047 (10)	−0.0087 (9)
O4	0.0302 (12)	0.0313 (12)	0.0218 (10)	−0.0179 (11)	0.0088 (9)	−0.0057 (9)

### Geometric parameters (Å, º)

**Table:**

Fe1—O5	2.121 (2)	C1—H1	0.9300
Fe1—O5^i^	2.121 (2)	N2—C2	1.377 (3)
Fe1—N1^i^	2.165 (2)	N2—H2A	0.8600
Fe1—N1	2.165 (2)	C2—C3	1.380 (3)
Fe1—O4^i^	2.1732 (16)	C2—C4	1.487 (3)
Fe1—O4	2.1732 (16)	C3—C5	1.489 (4)
O5—H1W	0.86 (3)	C4—O1	1.226 (3)
O5—H2W	0.79 (3)	C4—O2	1.335 (3)
N1—C1	1.317 (3)	O2—H2	0.8200
N1—C3	1.377 (3)	C5—O3	1.257 (3)
C1—N2	1.336 (3)	C5—O4	1.269 (3)
			
O5—Fe1—O5^i^	180.00 (11)	N1—C1—N2	111.5 (2)
O5—Fe1—N1^i^	87.70 (9)	N1—C1—H1	124.3
O5^i^—Fe1—N1^i^	92.30 (9)	N2—C1—H1	124.3
O5—Fe1—N1	92.30 (9)	C1—N2—C2	108.2 (2)
O5^i^—Fe1—N1	87.70 (9)	C1—N2—H2A	125.9
N1^i^—Fe1—N1	180.00 (16)	C2—N2—H2A	125.9
O5—Fe1—O4^i^	90.06 (7)	N2—C2—C3	105.0 (2)
O5^i^—Fe1—O4^i^	89.94 (7)	N2—C2—C4	119.4 (2)
N1^i^—Fe1—O4^i^	76.79 (7)	C3—C2—C4	135.6 (2)
N1—Fe1—O4^i^	103.21 (7)	N1—C3—C2	109.3 (2)
O5—Fe1—O4	89.94 (7)	N1—C3—C5	118.0 (2)
O5^i^—Fe1—O4	90.06 (7)	C2—C3—C5	132.7 (2)
N1^i^—Fe1—O4	103.21 (7)	O1—C4—O2	121.8 (2)
N1—Fe1—O4	76.79 (7)	O1—C4—C2	121.5 (2)
O4^i^—Fe1—O4	180.00 (12)	O2—C4—C2	116.7 (2)
Fe1—O5—H1W	107 (2)	C4—O2—H2	109.5
Fe1—O5—H2W	113 (3)	O3—C5—O4	124.3 (3)
H1W—O5—H2W	117 (3)	O3—C5—C3	119.6 (2)
C1—N1—C3	106.0 (2)	O4—C5—C3	116.1 (2)
C1—N1—Fe1	141.85 (18)	C5—O4—Fe1	116.99 (16)
C3—N1—Fe1	112.14 (17)		

Symmetry code: (i) −*x*, −*y*, −*z*.

### Hydrogen-bond geometry (Å, º)

**Table:**

*D*—H···*A*	*D*—H	H···*A*	*D*···*A*	*D*—H···*A*
O5—H1*W*···O3^ii^	0.86 (3)	1.86 (3)	2.705 (3)	165 (3)
O5—H2*W*···O4^iii^	0.79 (3)	1.95 (3)	2.702 (3)	158 (3)
N2—H2*A*···O1^iv^	0.86	1.95	2.767 (3)	157
O2—H2···O3	0.82	1.85	2.665 (3)	174

Symmetry codes: (ii) −*x*, −*y*−1, −*z*; (iii) *x*+1, *y*, *z*; (iv) −*x*+1, −*y*, −*z*−1.

## References

[bb1] Altomare, A., Burla, M. C., Camalli, M., Cascarano, G. L., Giacovazzo, C., Guagliardi, A., Moliterni, A. G. G., Polidori, G. & Spagna, R. (1999). *J. Appl. Cryst.* **32**, 115–119.

[bb2] Du, C.-J., Song, X.-H., Wang, L.-S. & Du, C.-L. (2011). *Acta Cryst.* E**67**, m997. 10.1107/S1600536811024779 PMC3151786 21836968

[bb3] Fang, R.-Q. & Zhang, X.-M. (2006). *Inorg. Chem.* **45**, 4801–4810. 10.1021/ic052099m 16749845

[bb4] Farrugia, L. J. (1997). *J. Appl. Cryst.* **30**, 565.

[bb5] Higashi, T. (1995). *ABSCOR* Rigaku Corporation, Tokyo, Japan.

[bb6] Li, J.-X., Du, Z.-X., Wang, L.-Z. & Huang, W.-P. (2011). *Inorg. Chim. Acta*, **376**, 479–485.

[bb7] Rigaku (1998). *RAPID-AUTO* Rigaku Corporation, Tokyo, Japan.

[bb8] Sheldrick, G. M. (2008). *Acta Cryst.* A**64**, 112–122. 10.1107/S0108767307043930 18156677

[bb9] Shimizu, E., Kondo, M., Ruwa, Y., Sarker, R. P., Miyazawa, M., Ueno, M., Naito, T., Maeda, K. & Uchida, F. (2004). *Inorg. Chem. Commun.* **7**, 1191–1194.

[bb10] Yakubovich, O. V., Karinova, O. V., Mel’nikov, O. K. & Urusov, V. S. (1995). *Dokl. Akad. Nauk*, **342**, 40–44.

